# Interleukin-1β promotes interleulin-6 expression via ERK1/2 signaling pathway in canine dermal fibroblasts

**DOI:** 10.1371/journal.pone.0220262

**Published:** 2019-07-25

**Authors:** Nanako Kitanaka, Rei Nakano, Kanae Sugiura, Taku Kitanaka, Shinichi Namba, Tadayoshi Konno, Tomohiro Nakayama, Hiroshi Sugiya

**Affiliations:** 1 Laboratory of Veterinary Biochemistry, Nihon University College of Bioresource Sciences, Kameino, Fujisawa, Kanagawa, Japan; 2 Laboratory for Cellular Function Conversion Technology, RIKEN Center for Integrative Medical Sciences, Suehiro-cho, Tsurumi, Yokohama, Kanagawa, Japan; 3 Laboratory of Veterinary Radiotherapy, Nihon University College of Bioresource Sciences, Kameino, Fujisawa, Kanagawa, Japan; Duke University School of Medicine, UNITED STATES

## Abstract

Interleukin-6 (IL-6) is a pleiotropic cytokine involved in the regulation of the immune response and inflammation. In this study, we investigated effect of the proinflammatory cytokine interleukin-1β (IL-1β) on IL-6 expression in canine dermal fibroblasts. IL-1β induced IL-6 mRNA expression and protein release in a time- and dose-dependent manner. When cells were treated with inhibitors of mitogen-activated protein kinases (MAPKs), the extracellular signal-regulated kinase (ERK) inhibitor FR180240 inhibited IL-1β-induced IL-6 mRNA expression, but not SP600125 or SKF86002, which are c-Jun N-terminal kinase (JNK) and p38 MAPK inhibitors, respectively. In cells treated with U0126, an inhibitor of MAPK/ERK kinase (MEK), which activates ERK, IL-1β-induced IL-6 mRNA expression was also inhibited. IL-1β stimulated ERK1/2 phosphorylation. In cells transfected with ERK1 and ERK2 isoform siRNAs, IL-1β-induced IL-6 mRNA expression was reduced. These observations suggest that IL-1β induces IL-6 expression via ERK1/2 signaling pathway in canine dermal fibroblasts.

## Introduction

Interleukin-6 (IL-6) is a pleiotropic cytokine involved in the regulation of the immune response and inflammation. In human, IL-6 is narrowly detectable in serum under physiological conditions, but its concentration dramatically increases during early phases of inflammation [1, 2].

In dogs with inflammation experimentally induced by an injection of turpentine oil [3] or canine sepsis models produced by administering infusions of either live *Escherichia coli* [4] or lipopolysaccharide (LPS) [5–7], induction of high levels of serum IL-6 are observed. Furthermore, in dogs with naturally occurring systemic inflammatory response syndrome (SIRS) and sepsis [8], joint inflammation caused by idiopathic immune-mediated polyarthropathy [9], or with metaphyseal osteopathy (MO), an inflammatory bone disease [10], high plasma IL-6 concentrations were observed. Therefore, IL-6 is likely a crucial cytokine for inflammatory process in dogs and humans.

In the skin, the increase in IL-6 expression and production is associated with inflammatory skin diseases, such as psoriasis [11, 12], lichen planus [13], systemic sclerosis [14], and systemic lupus erythematosus [15]. Overexpression of IL-6 in the skin of rats has been demonstrated to induce epidermal proliferation and inflammation [16]. IL-6 plays a crucial role in the pathogenesis of not only systemic but also local inflammation and is involved in the growth and differentiation of numerous cell types, including cells of dermal and epidermal origin in the skin [17].

MAPKs (mitogen-activated protein kinases) are key enzymes that participate in the signal transduction cascade from the extracellular environment to the nucleus of essentially every eukaryotic cell type and are involved in directing cellular response to a diverse array of stimuli including inflammatory cytokines [18]. MAPKs have three main pathways: the extracellular signal-regulated kinase (ERK) 1/2, c-jun N-terminal kinase (JNK), and p38 MAPK [18, 19].

Fibroblasts, a major cellular component of connective tissue, produce inflammatory cytokines and chemokines in response to numerous stress stimuli, such as bacterial endotoxins, cytokines and growth factors, and participate in the regulation of inflammatory reactions cooperatively with immune cells [20, 21]. Interleukin-1β is a potent pro-inflammatory cytokine that is involved in host immune and inflammatory responses [22]. IL-1β stimulates IL-6 expression and release via MAPK signaling pathways in various human and rat cells [23–31]. In this study, we demonstrate that IL-1β mediates IL-6 expression via ERK1/2 in canine dermal fibroblasts.

## Material and methods

### Materials

TRIzol was obtained from Life Technologies Co. (Carlsbad, CA). CELLBANKER 1 plus medium, PrimeScript RT Master Mix, SYBR Premix Ex Taq II, Thermal Cycler Dice Real Time System II and TP900 DiceRealTime v4.02B were obtained from TaKaRa Bio Inc. (Shiga, Japan). Rabbit monoclonal antibodies against human phospho-ERK1/2 (p-ERK1/2, D13.14.4E) and rat total-ERK1/2 (t-ERK1/2, 137F5) were purchased from Cell Signaling Technology Japan, K.K. (Tokyo, Japan). Horseradish peroxidase-conjugated (HRP-conjugated) anti-rabbit IgG antibody, ECL Western blotting Analysis System and ImageQuant LAS 4000 mini were purchased from GE Healthcare (Piscataway, NJ).

Polyvinylidene difluoride (PVDF) membranes and Mini-PROTEAN TGX gel were obtained from Bio-Rad (Hercules, CA). Block Ace and Complete mini EDTA-free protease inhibitor mixture were purchased from Roche (Mannheim, Germany). α-Modified Eagle minimum essential medium (α-MEM), phenylmethanesulfonyl fluoride (PMSF), sodium fluoride and 4-(2-hydroxyethyl)-1-piperazineethanesulfonic acid (HEPES) were purchased from Wako Pure Chemical Industries, Ltd. (Osaka, Japan). Canine IL-6 ELISA kit was purchased from R&D Systems, Inc. (Minneapolis, MN). StatMate IV was obtained from ATMS (Tokyo, Japan). Fetal bovine serum (FBS) was obtained from Biowest (France). U0126, FR180204, SKF86002 and SP600125 were purchased from Sigma-Aldrich Inc. (St Louis, MO). Canine recombinant IL-1β was purchased from Kingfisher Biotech, Inc. (Saint Paul, MN).

### Cell culture

Dermal fibroblasts were prepared from dorsal skin of three healthy beagle dogs (3-year-old male). This study was approved by Nihon University Animal Care and Use Committee (AP13B051). Skin samples were collected after local anesthesia with 1% lidocaine and 10 μg/mL adrenaline. To alleviate pain, butorphanol tartrate (0.2 mg/kg) was administered intravenously after the procedure. Canine dermal fibroblasts were isolated by explant culture using a method previously described [32, 33]. Briefly, canine dermis collected from the dorsal skin was cut into 3-mm^2^ sections. Each explant was placed into 90 mm Petri dish, and attached explants were maintained in a static-culture in an incubator at 5% CO_2_ and 37°C using α-MEM supplemented with 10% FBS. The medium was changed once a week, and canine dermal fibroblasts were obtained as outgrowth cells. Canine dermal fibroblasts were harvested using 0.25% trypsin-EDTA once they reached 90–95% confluence. The collected cells were suspended using CELLBANKER 1 plus medium at a density of 2 × 10^6^ cells/500 μL, and 500 μL of the cell suspension were placed into each sterilized serum tube. The tubes were then placed into a freezing vessel (BICELL; Nihon Freezer Co., Ltd., Tokyo, Japan) and cryopreserved at -80°C. Before experiments, serum tubes were removed from the BICELL vessel and immersed into a water bath at 37°C. The thawed-out cell suspension was transferred into a centrifuge tube with α-MEM containing 10% FBS and centrifuged at 300 g for 3 min. After removal of the supernatant, the pellet was suspended in α-MEM containing 10% FBS and transferred into a 75-cm^2^ culture flask. Static cultures were then maintained under the same conditions as used before the cryopreservation. Cells were harvested using 0.25% trypsin-EDTA once they reached approximately 90% confluence. Then, the collected cells were seeded at a density of 1 × 10^6^ cells per 75-cm^2^ culture flask. Fourth passage dermal fibroblasts were used for the following experiments. Cells from different animals were used in different experiments.

### Real-time RT-PCR

Total RNA was extracted from dermal fibroblasts with TRIzol reagent. First-strand cDNA synthesis was performed with 500 ng of total RNA using PrimeScript RT Master Mix. Real-time RT-PCR was performed with 2 μL of the first-strand cDNA in 25 μL (total reaction volume) with SYBR Premix Ex Taq II and primers specific for canine IL-6 and TATA box binding protein (TBP), a house keeping protein used as a control. Table 1 shows primer sequences used for real-time RT-PCR. Real-time RT-PCR of no-template controls was performed with 2 μL RNase- and DNA-free water. In addition, real-time PCR of no-reverse transcription control was performed with 2 μL of each RNA sample. PCR was conducted using Thermal Cycler Dice Real Time System II with the following protocol: 1 cycle of denaturing at 95°C for 30 s, 40 cycles of denaturing at 95°C for 5 s and annealing/extension at 60°C for 30 s. The results were analyzed by the second derivative maximum method and the comparative cycle threshold (ΔΔCt) method using real-time RT-PCR analysis software. Amplification of TBP from the same amount of cDNA was used as an endogenous control, while cDNA amplification from canine dermal fibroblasts at time 0 was used as a calibration standard.

**Table 1:** Primers used for real-time RT-PCR.

Gene Name	Gene bank ID	Primer sequences
*IL-6*	NM_001003301.1	F: 5ʹ- CAAGATCCTGGTCCAGATGCTAAAG-3ʹ
		R: 5ʹ- CACTCATCCTGCGACTGCAA-3ʹ
*TBP*	XM_863452	F: 5′-ACTGTTGGTGGGTCAGCACAAG-3′
		R: 5′-ATGGTGTGTACGGGAGCCAAG-3′

### Western blotting

The cells were lysed with a lysis buffer containing 20 mM HEPES, 1 mM PMSF, 10 mM sodium fluoride, and complete mini EDTA-free protease inhibitor cocktail at pH 7.4. Protein concentrations were adjusted using the Bradford method [34]. Extracted proteins were boiled at 95°C for 5 min in SDS buffer. Samples were loaded into separate lanes of 12% Mini-PROTEAN TGX gel and electrophoretically separated. Separated proteins were transferred to PVDF membranes, treated with Block Ace for 50 min at room temperature, and incubated with primary antibodies [p-ERK1/2 (1:1000), t-ERK1/2 (1:1000)] for 120 min at room temperature. After washing, the membranes were incubated with an HRP-conjugated anti-rabbit or a mouse IgG antibody (1:10000) for 90 min at room temperature. Immunoreactivity was detected using ECL Western Blotting Analysis System. Chemiluminescent signals of the membranes were measured using ImageQuant LAS 4000 mini.

### siRNA transfection

Canine dermal fibroblasts seeded at a density of 1 × 10^5^ cells/35-mm dish or 5 × 10^5^ cells/90 mm dish, were transfected using Opti-MEM containing 5 μL/mL Lipofectamine 2000 and 400 nM ERK1, ERK2 or scrambled siRNA for 6 h (Nakano et al., 2018). The siRNA sequences are indicated in Table 2. The efficiency of siRNA was confirmed by western blotting.

**Table 2:** Sequences used for siRNA transfection.

Gene Name	Gene bank ID	siRNA sequences
*ERK1*	NM_001252035.1	F: 5'-CCAAUGUGCUCCACCGGGA-3'
		R: 5'-UCCCGGUGGAGCACAUUGG-3'
*ERK2*	NM_001110800.1	F: 5'-CCCAAAUGCUGACUCGAAA-3'
		R: 5'-UUUCGAGUCAGCAUUUGGG-3'

### IL-6 assay

Canine dermal fibroblasts were seeded at a density of 3 × 10^5^ cells per well in 6-well culture plates. The cells were treated with IL-1β, and culture supernatants were collected. The concentration of IL-6 in the culture supernatant was measured using an IL-6 ELISA kit according to the manufacturer’s instructions.

### Statistical analysis

The data from these experiments are presented as the mean ± standard error of measurement. Statistical analysis was performed using StatMate IV. The data from the time course study were analyzed using two-way analysis of variance, and the data from other experiments were analyzed using one-way analysis of variance. Tukey’s test was used as post hoc analysis. *P*-values less than 0.05 were considered statistically significant.

## Results

### IL-1β induced IL-6 production in canine dermal fibroblasts

**Fig 1:**
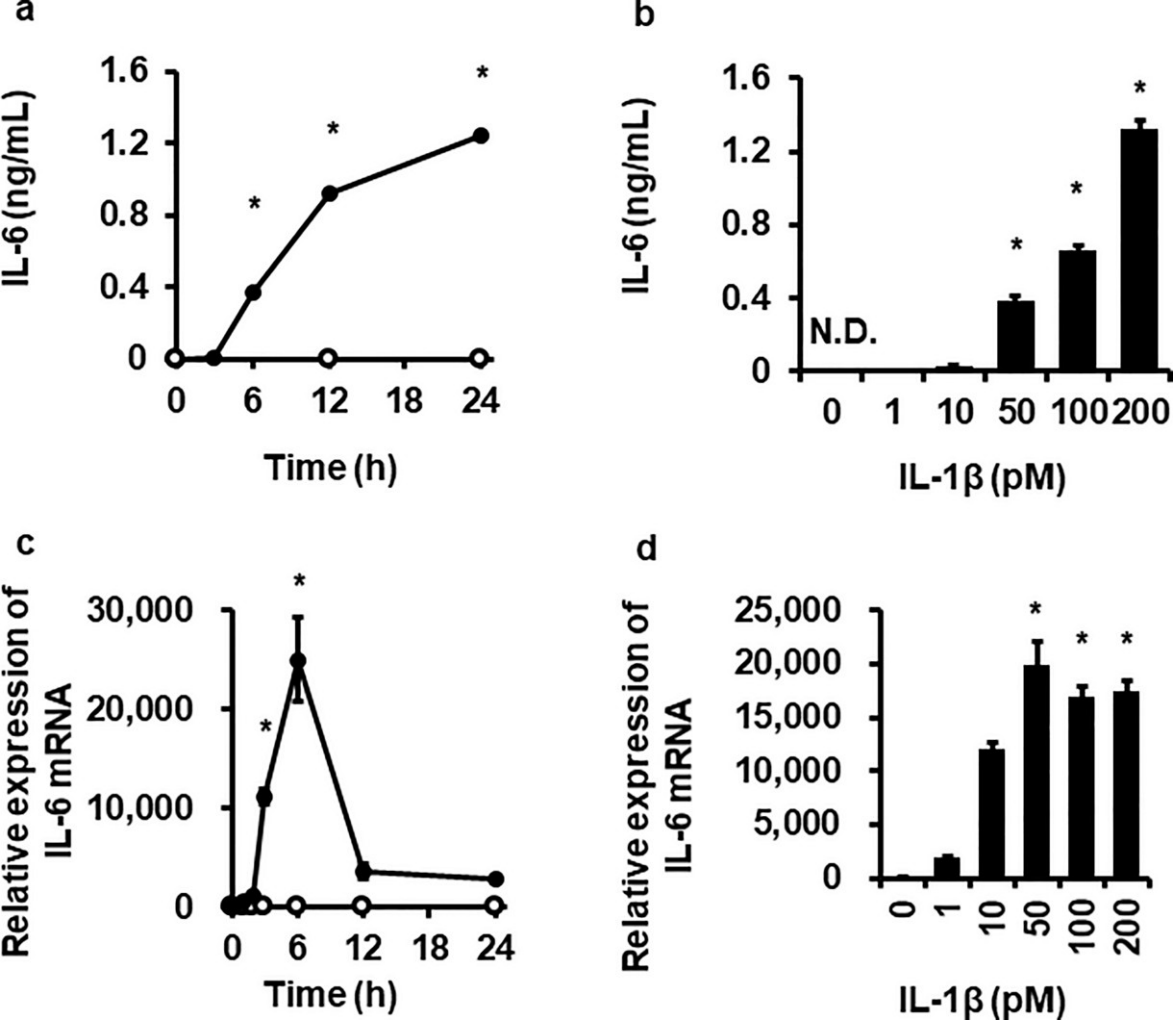
Time- and dose-dependent IL-1β-induced IL-6 protein release and mRNA expression in canine dermal fibroblasts. The cells were incubated with (closed circle) or without (open circle) 200 pM IL-1β for indicated times (a, c) or with indicated concentrations of IL-1β for 24 h (b) or 6 h (d). At the end of the incubation, protein release and mRNA expression of IL-6 were detected by ELISA and real-time RT-PCR, respectively. TBP was used as an internal standard. Values are expressed as the mean ± SE of 3 independent experiments. *P < 0.05, compared with 0 h (a, c), 0 pM (b, d).

We first examined the effect of IL-1β on IL-6 release in canine dermal fibroblasts. When the cells were exposed to 200 pM IL-1β for 0–24 h, a significant increase in IL-6 levels released into culture media was observed from 6 h to 24 h in a time-dependent manner (Fig 1A). In cells treated with 0–200 pM IL-1β for 24 h, a dose-dependent increase in IL-6 release was observed with dose levels between 50 and 200 pM (Fig 1B). Then we examined the effect of IL-1β on IL-6 mRNA expression. After treatment with 100 pM IL-1β, a significant increase in IL-6 mRNA expression significantly increased at 3 h, reached a peak level at 6 h, and then returned to the sustained levels that were slightly higher than the control (Fig 1C). In the cells exposed to 0–200 pM IL-1β for 6 h, a significant increase in IL-6 mRNA expression was observed with dose levels between 50 and 200 pM (Fig 1D). These observations indicate that IL-1β mediates IL-6 production in canine dermal fibroblasts. Since effect of IL-1β on IL-6 mRNA expression reached a plateau at over 50 pM, 100 pM of IL-1β were used for all the following experiments.

### Involvement of the ERK1/2 pathway in IL-1β-induced IL-6 production

**Fig 2:**
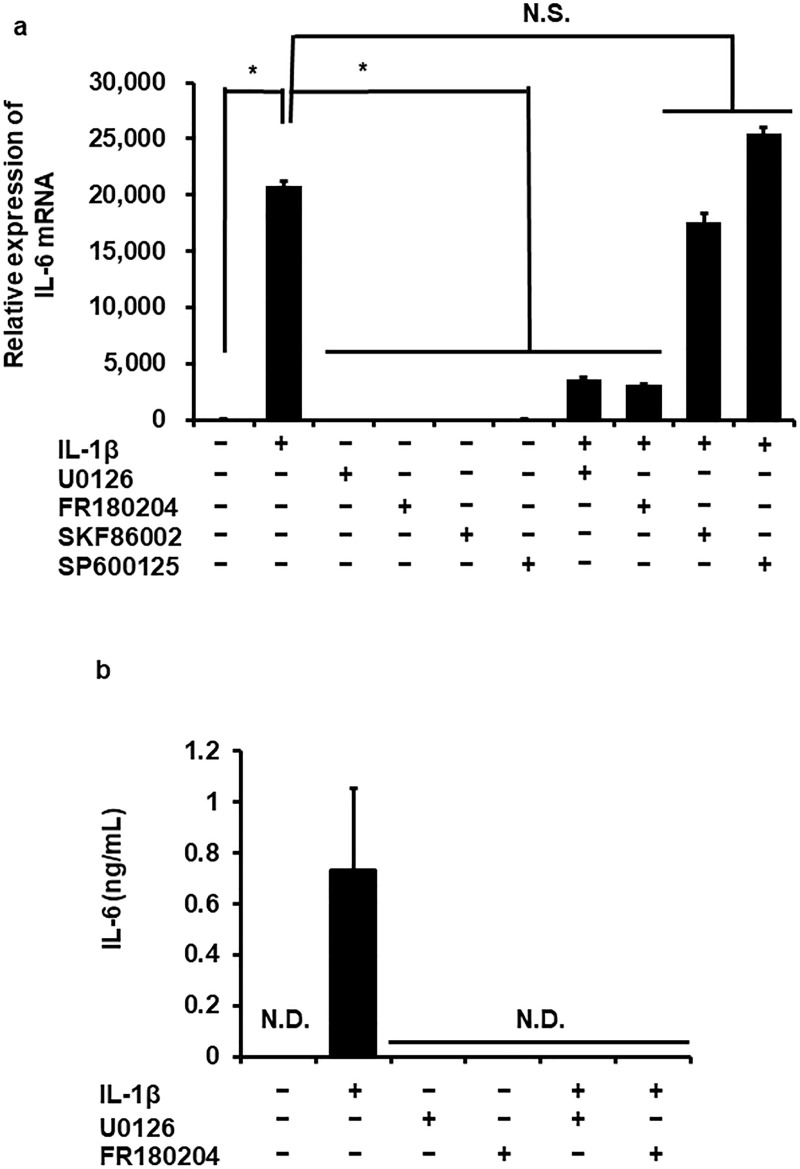
Inhibitory effect of ERK1/2 and MEK inhibitors on IL-1β-induced IL-6 mRNA expression and release. After the pretreatment without (control) or with FR180204 (25 μM), SKF86002 (20 μM), SP600125 (10 μM) and U0126 (10 μM) (ERK1/2, p38 MAPK, JNK and MEK inhibitors, respectively) for 1 h, and then fibroblasts were stimulated with 100 pM IL-1β for 6 h (a) or 24 h (b). ERK1/2 and MEK inhibitors attenuated IL-1β-induced IL-6 mRNA expression (a) and protein release (b) but not p38 MAPK and JNK inhibitors. TBP was used as an internal standard (a). Values are expressed as the mean ± SE of 3 independent experiments. *P < 0.05, compared with control.

To evaluate the involvement of MAPK signaling pathways in IL-1β-induced IL-6 production, we determined the effect of pharmacological MAPK inhibitors on IL-1β-induced IL-6 mRNA expression. Cells were pretreated with FR180204 (25 μM), SKF86002 (20 μM) or SP600125 (10 μM) (ERK1/2, p38 MAPK or JNK inhibitors, respectively) for 1 h and then stimulated with 100 pM IL-1β for 6 h. As Fig 2A summarizes, the ERK1/2 inhibitor FR180204 clearly inhibited IL-1β-induced IL-6 mRNA expression, but the p38 MAPK inhibitor SKF86002 or the JNK inhibitor SP600125 did not. ERK1/2 is activated by MAPK/ERK kinase (MEK) [18]. Then we examined the effect of the MEK inhibitor U0126. In cells pretreated with U0126 (10 μM) for 1 h, the effect of IL-1β on IL-6 mRNA expression was significantly reduced (Fig 2A). In cells pretreated with FR180204 or U0126, IL-1β failed to induce IL-6 release, as shown in Fig 2B. These observations suggest that the ERK1/2 signaling pathway is involved in IL-1β-induced IL-6 production.

### IL-1β-induced ERK1/2 phosphorylation

**Fig 3:**
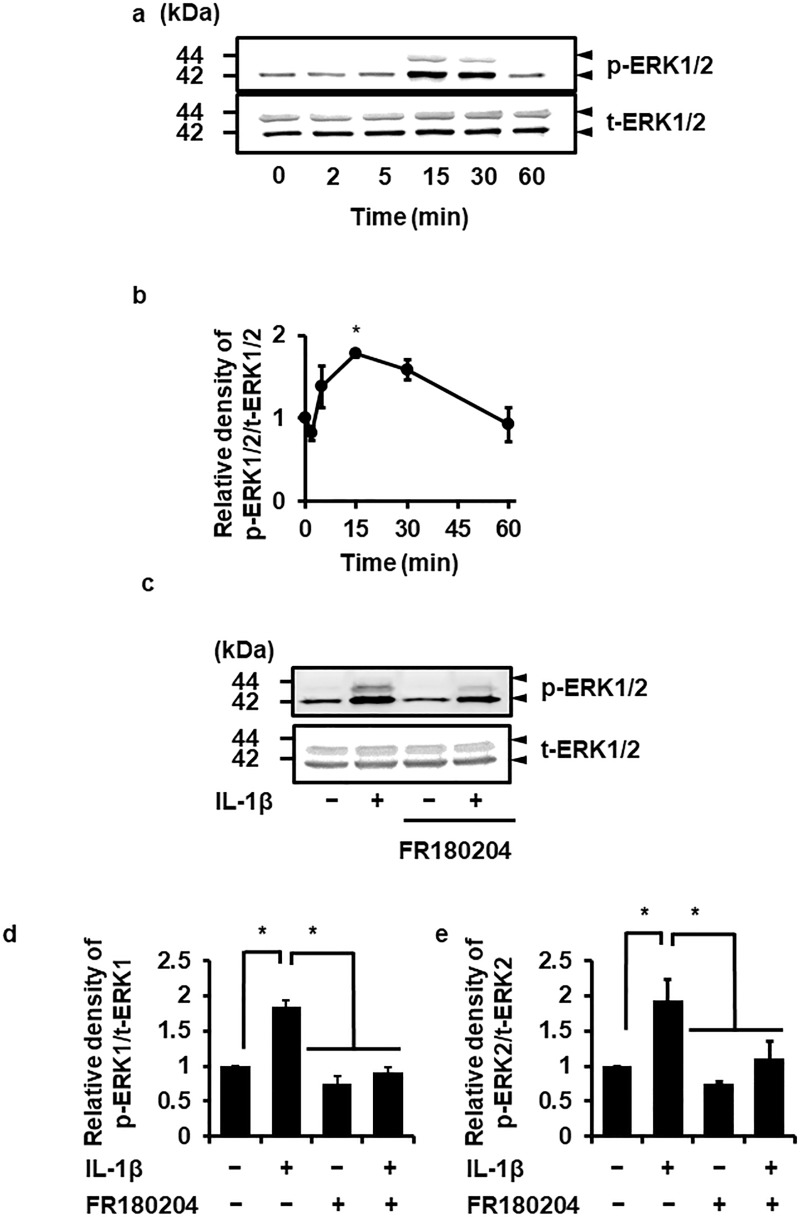
IL-1β-induced ERK1/2 phosphorylation and its inhibition by an ERK1/2 inhibitor. In dermal fibroblasts treated with 100 pM IL-1β for 0–60 min, ERK1/2 phosphorylation (p-ERK1/2) was observed in a time-dependent manner (a, b). IL-1β had no effect on total ERK1/2 (t-ERK1/2) expression (a). In cells pretreated without (control) or with the ERK1/2 inhibitor FR180204 (25 μM) for 1 h, IL-1β-induced ERK1/2 phosphorylation was attenuated (c, d). Representative results (a, c) and the relative density of ERK1/2 phosphorylation compared with the results at 0 time (b) or the control (d) are illustrated. Values are expressed as the mean ± SE of 3 independent experiments (b, d). *P < 0.05.

Since ERK1/2 is activated by its phosphorylation [18, 35], we determined IL-1β-induced ERK1/2 phosphorylation in canine dermal fibroblasts. Fig 3A and 3B summarize time-dependent ERK1/2 phosphorylation in cells exposed to IL-1β (100 pM) for 0–60 min. ERK1/2 phosphorylation occurred transiently and peaked at 15 min. However, IL-1β failed to induce the phosphorylation of JNK and p38 (S1 Fig). IL-1β-induced ERK1/2 phosphorylation was attenuated in cells pretreated with the ERK1/2 inhibitor FR180204, as shown in Fig 3C, 3D and 3E. Taken together, ERK1/2 is likely involved in IL-1β-induced IL-6 expression.

### Attenuation of IL-1β-induced IL-6 mRNA expression in ERK1 and ERK2-knockdown cells

**Fig 4:**
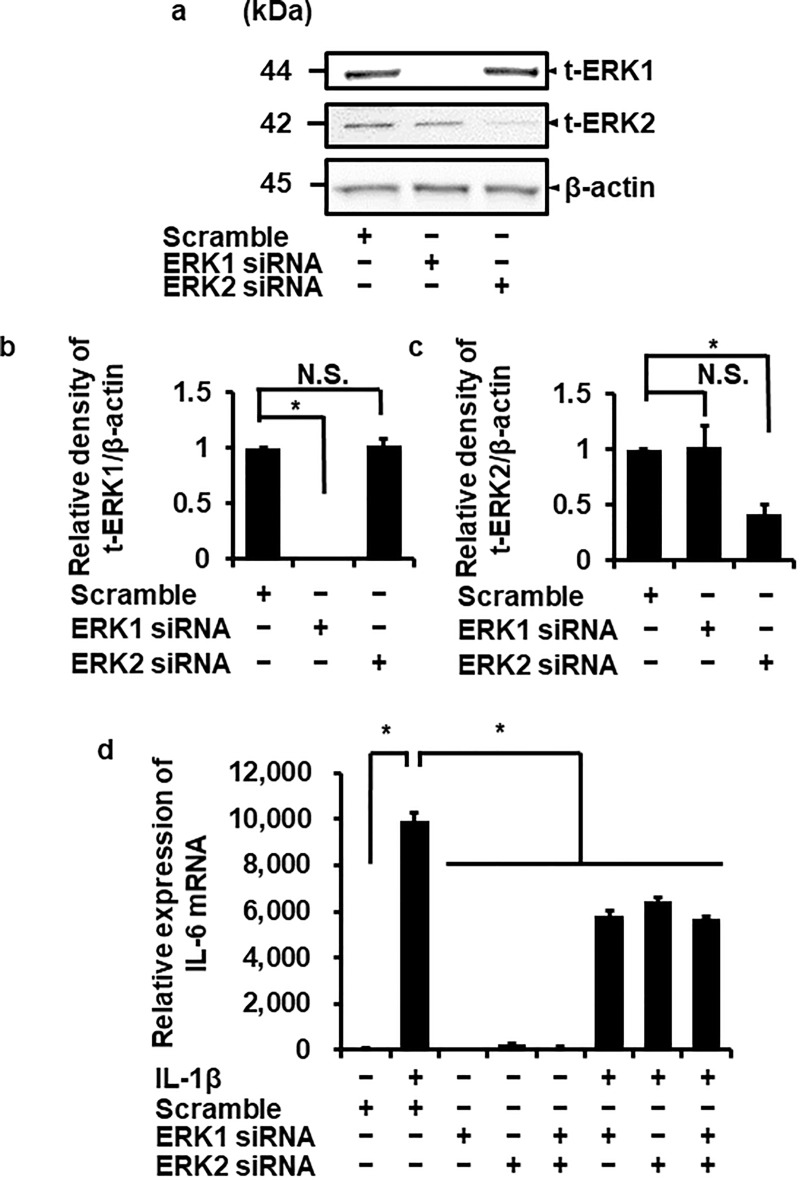
Attenuation of IL-1β-induced IL-6 mRNA expression in canine dermal fibroblasts transfected with ERK1 and ERK2 siRNAs. (a-c) In fibroblasts transfected with ERK1, ERK2, and scrambled siRNAs, expression of t-ERK1, t-ERK2, and β-actin was detected by western blotting. ERK1 or ERK2 siRNA transfection decreased the expression of ERK1 or ERK2, respectively, while scrambled siRNA transfection did not influence their expression. β-actin was used as an internal standard. Representative results (a) and relative density of ERK1 or ERK2 protein expression in siRNA-transfected cells compared with those in scrambled siRNA-transfected cells (b, c) are illustrated. (d) After transfection with ERK1, ERK2 and scrambled siRNAs, fibroblasts were incubated with or without 100 pM IL-1β for 6 h. At the end of the incubation, IL-6 mRNA expression was determined. TBP was used as an internal standard. ERK1 and ERK2 siRNA transfection reduced IL-1β-induced IL-6 mRNA expression while scrambled siRNA-transfection did not. IL-1β-induced IL-6 mRNA expression was also reduced in ERK1 and 2 double knockdown cells. Values are expressed as the mean ± SE of 3 independent experiments. *P < 0.05.

To confirm the involvement of ERK1/2 in IL-1β-induced IL-6 mRNA expression, we performed ERK1/2 knockdown experiment using siRNA transfection. ERK1 or ERK2 protein expression was significantly reduced in cells transfected with ERK1 or ERK2 siRNAs, respectively, but not with scramble siRNA as a control (Fig 4A–4C). IL-1β-induced IL-6 mRNA expression was partially reduced in ERK1 and ERK2 siRNA-transfected cells compared with the scramble siRNA-transfected cells (Fig 4D). In ERK1 and 2 double-knockdown cells, IL-1β-induced IL-6 mRNA expression was also attenuated, but the reduction level was the same as that in ERK1 and ERK2 siRNA-transfected cells (Fig 4D). These observations suggest that the ERK1/2 activation contributes to the upregulation of IL-6 mRNA expression induced by IL-1β in canine dermal fibroblasts.

## Discussion

Wound healing is a highly coordinated and interactive process involving several overlapping stages that include inflammation, formation of granulation tissue, re-epithelization, matrix formation and remodeling [36]. The interaction of different cell types including keratinocytes, fibroblasts, endothelial cells, macrophages, and platelets is involved in the induction of a sequence of such events. Dermal fibroblasts are important cells in cutaneous wound healing processes through their proliferation, ordered migration into the provisional matrix, production of extracellular matrix and differentiation into myofibroblasts [21, 37]. In the present study, we demonstrated that IL-1β stimulates IL-6 production and release in canine dermal fibroblasts.

IL-6 is readily detected in mouse skin wounds [38]. In human, IL-6 is produced and released rapidly after full thickness skin wounding and persisted even up to 24 h after injury [39]. In mice genetically deficient in IL-6, wound healing is significantly delayed with attenuated leukocyte infiltration, re-epithelialization, angiogenesis and collagen accumulation [40]. In a subsequent study, administration of a neutralizing anti-IL-6 monoclonal antibody significantly delays wound closure in normal mice [40]. A chimeric fusion protein consisting of IL-6 and soluble IL-6 receptor termed ‘Hyper-IL-6’ accelerates skin wound healing in a mouse skin damage model [41]. IL-6 induces keratinocytes migration through the production of a soluble fibroblast-derived factor [42]. These observations clearly indicate that IL-6 is a major regulator of the skin wound healing.

IL-1β, a proinflammatory cytokine, plays a pivotal role in the initiation and amplification of inflammation in various tissues. IL-1β is produced primarily by macrophages and monocytes, as well as by nonimmune cells including activated fibroblasts and keratinocytes, which contributes to wound healing including that noted in the skin [43, 44]. IL-1 derived from keratinocytes has been demonstrated to induce the production of cytokines including IL-6 in fibroblasts [45, 46]. Therefore, it is likely that IL-1β-induced IL-6 production is a pivotal process in wound healing in dog skin.

IL-1β provokes IL-6 production and release via MAPK signaling pathways in various cells, for example, p38 MAPK in human retinal Müller cells [23] and rheumatoid fibroblast-like synoviocytes [24–26]; p38 and ERK1/2 in human orbital fibroblasts [27], chondrocyte cell line C-28/I2 [28] and gingival fibroblasts [29]; p38 and JNK in rat glial cells [30]; and ERK1/2 in rat synovial fibroblasts [31] contribute to IL-1β-induced IL-6 expression and production. These studies imply that MAPK pathway involvement in IL-1β-induced IL-6 expression is depends on the cell type and species. In this study, ERK1/2 and MEK inhibitors attenuated the effect of IL-1β on IL-6 mRNA expression, but p38 and JNK inhibitors did not. In ERK1- and ERK2-knockdown cells, IL-1β-induced IL-6 expression was reduced. Therefore, it is likely that the ERK1/2 signaling pathway is dominantly involved in IL-1β-induced IL-6 expression in canine dermal fibroblasts.

Human ERK1 and ERK2 are 84% identical and are coexpressed in most tissues [35, 47]. Coactivation of these two isoforms generally occurs in cells stimulated with multiple extracellular stimuli [48–50]. On the other hand, functional differences between the two isoforms were observed [51–55]. We also demonstrated that ERK1 and ERK2 have different functions in feline and canine synovial fibroblasts [56, 57] and canine dermal fibroblasts [33]. Then, we performed ERK-knockdown experiments by treatment with ERK isoform-specific siRNA. IL-1β-induced IL-6 mRNA expression was attenuated both in ERK1- and ERK2-knockdown cells. To confirm the compensation of ERK1 and ERK2 pathways, we examined the effect of IL-1β on IL-6 mRNA expression in the cells co-transfected with both ERK1 and ERK2 siRNAs. In the co-transfected cells, IL-1β-induced IL-6 mRNA expression was reduced compared with control. However, the reduction by the co-transfection was no different from that by the single transfection with ERK1 or ERK2 siRNA. These observations suggest that the functions of ERK1 and ERK2 are identical. Therefore, it is unlikely that there is compensation mechanism between ERK1 and ERK2, although further studies need to clarify the relations between ERK1 and ERK2 in canine dermal fibroblasts.

IL-1β-induced IL-6 mRNA expression was partially reduced in ERK1/2- knockdown cells, whereas the effect was completely inhibited in cells treated with an ERK1/2 inhibitor. Eight isoforms of ERK are present [35, 58]. In the present study, we cannot exclude the possibility of the contribution of the other ERK isoforms in IL-1β-induced IL-6 mRNA expression. Although FR180204 has been a widely used specific inhibitor for ERK1/2, the inhibitor appears to influence the other isoforms. Studies with the other isoforms of ERK on IL-1β-induced IL-6 expression are underway in our laboratory.

## Conclusions

In conclusion, we demonstrated that IL-1β induced IL-6 expression via the ERK1/2 signaling pathway in canine dermal fibroblasts using pharmacological inhibitors and ERK1/2-knockdown cells. Since MAPK signaling pathways are molecular targets for anti-inflammatory therapy [59], it is likely that the ERK1/2 signaling pathway could represents a target for therapy for skin inflammation in dogs.

## Supporting information

S1 Fig No effect of IL-1β on the phosphorylation of JNK and p38. The levels of phosphorylated JNK (p-JNK), total JNK (t-JNK), phosphorylated p38 (p-p38) and total p38 (t-p38) were detected by western blotting in dermal fibroblasts treated with 100 pM IL-1β for 0–120 min. IL-1β failed to activate JNK and p38. Results are representative in three independent experiments. Canine dermal fibroblasts from three beagle dogs were used, and each experiment was performed with cells derived from a single donor.
(PPTX)
