# Supplementary material for: Interleukin-1β promotes interleulin-6 expression via ERK1/2 signaling pathway in canine dermal fibroblasts
Source: PLoS One. 2019 Jul 25;14(7):e0220262. doi: 10.1371/journal.pone.0220262 (PMC6658082; doi:10.1371/journal.pone.0220262)
Supplement: S1 Fig — The levels of phosphorylated JNK (p-JNK), total JNK (t-JNK), phosphorylated p38 (p-p38) and total p38 (t-p38) were detected by western blotting in dermal fibroblasts treated with 100 pM IL-1β for 0–120 min. IL-1β failed to activate JNK and p38. Results are representative in three independent experiments. Canine dermal fibroblasts from three beagle dogs were used, and each experiment was performed with cells derived from a single donor. (PPTX) [file pone.0220262.s001.pptx]

## Slide 1
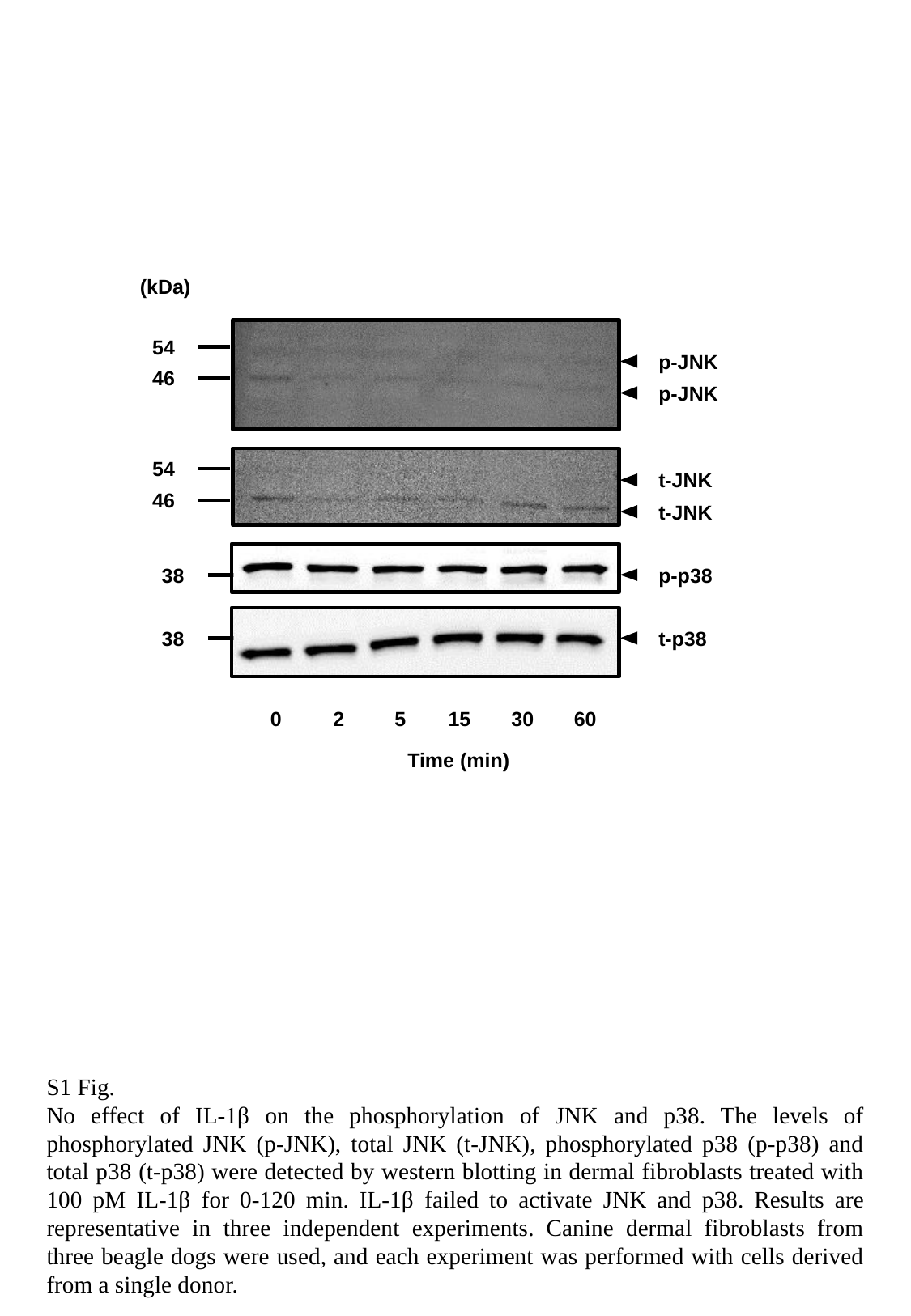

(kDa)
54
p-JNK
46
p-JNK
54
t-JNK
46
t-JNK
38
p-p38
38
t-p38
0
2
5
15
30
60
Time (min)
S1 Fig.
No effect of IL-1β on the phosphorylation of JNK and p38. The levels of phosphorylated JNK (p-JNK), total JNK (t-JNK), phosphorylated p38 (p-p38) and total p38 (t-p38) were detected by western blotting in dermal fibroblasts treated with 100 pM IL-1β for 0-120 min. IL-1β failed to activate JNK and p38. Results are representative in three independent experiments. Canine dermal fibroblasts from three beagle dogs were used, and each experiment was performed with cells derived from a single donor.

## Slide 2
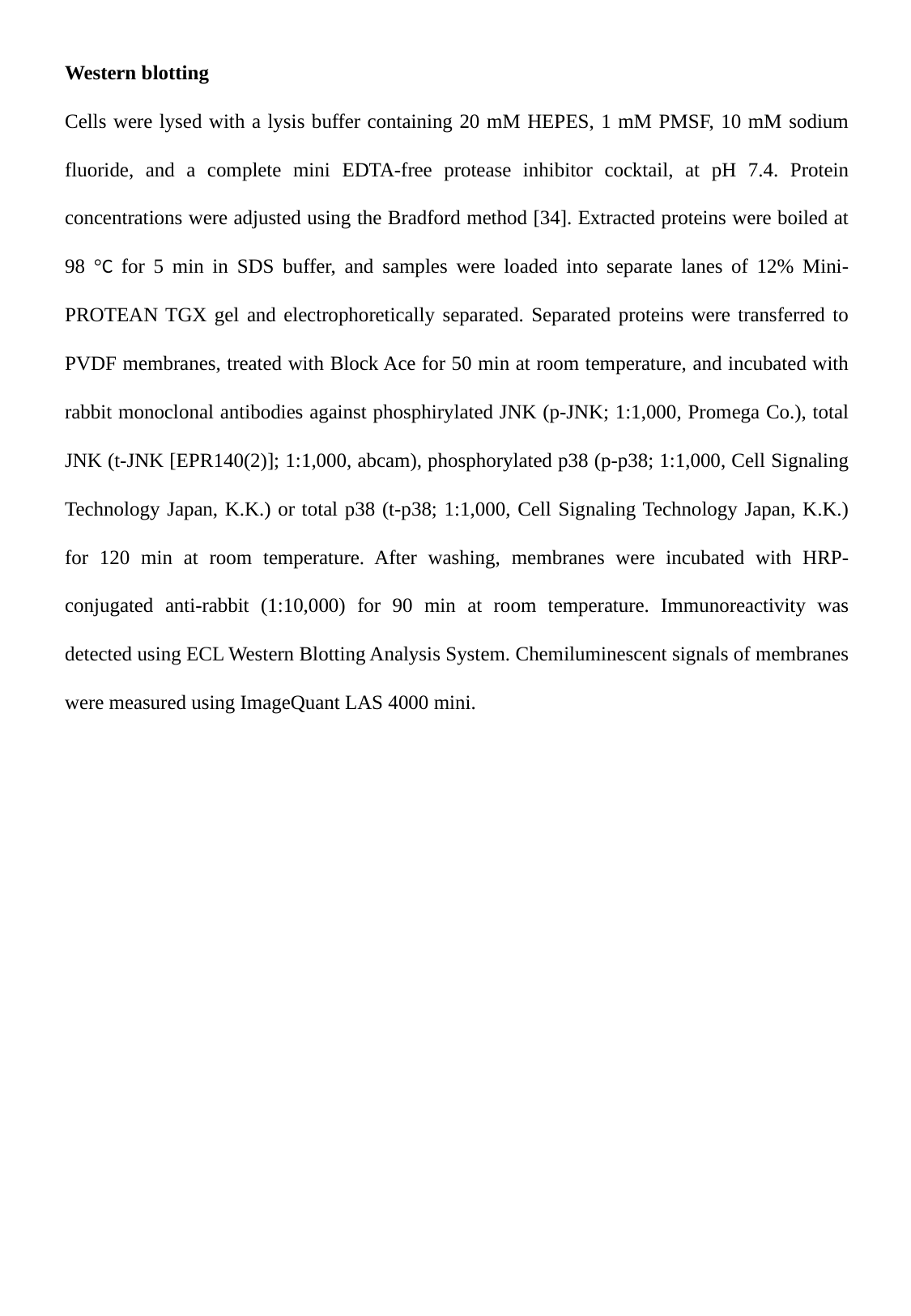

Western blotting
Cells were lysed with a lysis buffer containing 20 mM HEPES, 1 mM PMSF, 10 mM sodium fluoride, and a complete mini EDTA-free protease inhibitor cocktail, at pH 7.4. Protein concentrations were adjusted using the Bradford method [34]. Extracted proteins were boiled at 98 °C for 5 min in SDS buffer, and samples were loaded into separate lanes of 12% Mini-PROTEAN TGX gel and electrophoretically separated. Separated proteins were transferred to PVDF membranes, treated with Block Ace for 50 min at room temperature, and incubated with rabbit monoclonal antibodies against phosphirylated JNK (p-JNK; 1:1,000, Promega Co.), total JNK (t-JNK [EPR140(2)]; 1:1,000, abcam), phosphorylated p38 (p-p38; 1:1,000, Cell Signaling Technology Japan, K.K.) or total p38 (t-p38; 1:1,000, Cell Signaling Technology Japan, K.K.) for 120 min at room temperature. After washing, membranes were incubated with HRP-conjugated anti-rabbit (1:10,000) for 90 min at room temperature. Immunoreactivity was detected using ECL Western Blotting Analysis System. Chemiluminescent signals of membranes were measured using ImageQuant LAS 4000 mini.
